# Jute Fibers Synergy with nZVI/GO: Superficial Properties Enhancement for Arsenic Removal in Water with Possible Application in Dynamic Flow Filtration Systems

**DOI:** 10.3390/nano12223974

**Published:** 2022-11-11

**Authors:** Alejandra Moreno-Bárcenas, Jesús Alejandro Arizpe-Zapata, Julio Alejandro Rivera Haro, Pamela Sepúlveda, Alejandra Garcia-Garcia

**Affiliations:** 1 Advanced Materials Research Center, Design and Synthesis of Nanostructures and Bidimensional Materials Group, Apodaca 66628, NL, Mexico; 2 FCQ, Autonomous University of Nuevo León, San Nicolás de los Garza 64570, NL, Mexico; 3 Faculty of Chemistry and Biology and Faculty of Science, Physics Department, University of Santiago of Chile (USACH), Santiago 9170022, Chile; 4 Centro para el Desarrollo de Nanociencia y Nanotecnología CEDENNA, Santiago 9170022, Chile

**Keywords:** jute fibers, nZVI, graphene oxide, arsenic removal, iron nanoparticles

## Abstract

Groundwater is one of the primary sources of water for both drinking and industrial use in northeastern Mexican territory, around 46% of the total, due to the lack of precipitation during the year and solar radiation index. The presence of arsenic in brackish soil and groundwater is a severe health issue, specifically in semi-arid and arid regions in the north of Mexico. Additionally, it represents the only source of drinking water in communities far from big cities, mainly due to the absence of hydric infrastructure. This work presents a new approach to treating polluted water with arsenic. The system based on activating jute fiber with nanoparticles of zero-valent iron immobilized over graphene oxide will allow nZVI particles to preserve their unique qualities for water sanitization. A dynamic flow test was designed to determine the effectivity of activated jute fibers as a water sanitation system. The results showed a reduction in the total arsenic content from 350 ppb to 34 ppb with a filtrate flow of 20 mL/min. The above represents 90% adsorption by the activated fiber. The analyzed sample corresponds to contaminated groundwater taken from Coahuila, Mexico. This sanitation system could be applied to low-income populations lacking robust infrastructure, such arsenic treatment plants.

## 1. Introduction

The contamination of water sources for human consumption is a problem of great importance. Arsenic (As) is one of the contaminants present in nature, and it occupies the 20th place in terms of abundance, and its mobility extends to acidic or basic conditions. It is known as one of the most toxic elements and is identified as carcinogenic to humans. The intake of this metalloid could cause gastrointestinal and cardiovascular disorders, nervous system damage, and death [1,2]. The primary sources of arsenic contamination are industrial and mining effluents. They also occur due to natural causes such as diffusion in groundwater and thermal water sources. In countries worldwide, the most critical case is Bangladesh, millions of people are at risk of drinking water with arsenic contamination [3]. However, some states in the north of Mexico also have this problem, such as Coahuila.

Oxidation, precipitation/coprecipitation, coagulation, ion exchange, reverse osmosis, and electrokinetic methods have been widely employed to remove As from water [4]. Still, they have several drawbacks: high operating and waste treatment costs, high consumption of reagents, and a large volume of sludge formation [5]. The adsorption process is another widely reported process for As removal. The absorption is based on a superficial phenomenon between the adsorbate and the adsorbent by chemical interactions. It is recognized as an effective approach due to the low cost, high concentration efficiency, and environmental friendliness [6]. Some adsorbents in these processes include activated carbon, alumina, and iron oxide-based sorbents [7]. However, adsorbents and conventional techniques are often financially unviable for developing countries. Hence, natural adsorptive materials which are locally available offer sustainable and cost-effective solutions for the remediation of As pollution in low-income countries [1,8,9].

Therefore, an alternative is the use of natural fibers. For example, the modification of microcellulose fibers with iron nanoparticles for As adsorption reached a percentage of As removal of 67% in a water solution prepared with 0.68 mmol/L [10]. Cellulose fibers have also been applied as a support for MoS_2_ nanosheets allowing easy access to all fiber sites to carry out the adsorption of contaminants [11]. Chang-Gu et al. reported using amine-doped acrylic fibers for As adsorption. The fibers were prepared with dibasic sodium arsenate heptahydrate (Na_2_HAsO_4_·7H_2_O) and hydrochloric acid, and hydroxide solutions were used for pH adjustment. The tests showed an As removal percentage of 83% [12]. Another option is jute, a high-production natural fiber with industrial applications [13,14]. Jute is a biodegradable fiber; it is easy to produce in hot areas [15] such as India, China and Bangladesh [16,17] where the most critical problems due to As contamination occur. Jute is resistant and is used to reinforce composite materials. It contains 60 to 70% cellulose and has been used as a support for nanoparticles, allowing the nanometric material to anchor and distribute itself on its surface, reducing its agglomeration and increasing its specific surface area [18]. 

On the other hand, nanoscale zero-valent iron (nZVI) nanoparticles have shown improvements in the removal of a series of contaminants in aqueous solutions, including As [19,20,21]. The biggest problem with the addition of nanoparticles to a system is that a material with nanometric size forms agglomerates [22], diminishing its removal capacity. The combination of nZVI and graphene oxide (GO) represents an exciting proposal since it allows for a better nanoparticle disposition, reducing the agglomeration problems presented by nanomaterials.

In the present investigation, an approach is given that takes advantage of the characteristics of jute fiber to be activated with a hybrid nZVI/GO. The originality and novelty of this study lie in showing the synergy between the mentioned components, forming the hybrid compound of jute (J) and nZVI/GO (J-nZVI/GO) for the removal of As present in a sample of contaminated groundwater extracted from a well located in the Coahuila state in Mexico. Another important aspect is that the tests were performed in a dynamic flow, unlike the literature where adsorption tests are reported in batches [9]. The hybrid adsorbent was tested in a vertical filtration column. In the proposed arrangement, the force of gravity exerts its influence to carry out the filtration. The system was designed for its application in low-resource areas where infrastructure such as treatment plants is lacking. The hybrid compound (J-nZVI/GO) was analyzed by scanning electron microscopy (SEM), energy dispersive X-ray spectroscopy (EDS), and Fourier transform infrared spectroscopy (FTIR) before and after filtration. The water sample was studied before and after the filtering process by plasma emission spectrometry (ICP-OES). Finally, J-nZVI/GO was treated for reactivation.

## 2. Materials and Methods

Natural jute fiber, iron nitrate (Fe(NO_3_)_3_·9H_2_O), sodium borohydride (NaBH_4_), hydrochloric acid (HCl), sodium hydroxide (NaOH), ultra-pure argon gas (Ar), and graphite powder were utilized, all were bought from Sigma Aldrich (Rahway, NJ, USA). For the test batch, we used well water from the state of Coahuila, Mexico (supplied by AQUAMEX company). The real contaminated groundwater has 350 µg/L of total arsenic at pH 7. It was determined by ICP-OES, ICAP 6500 duo (Thermo Electron Corporation, Cambridge, UK). The equipment used in the morphology characterization was a NOVA NANOSEM200 microscope (FEI, Hillsboro, OR, USA) for the nanoparticles and a Nanotech TEM JEOL 6010 plus (JEOL Akishima Tokyo, Japan) with an EDS (energy dispersive spectroscopy) detector and a Nicolet IS50 FTIR spectrometer (Thermo Fisher Scientific, Waltham, MA, USA) ATR mode for the fibers.

### 2.1. Synthesis: GO, nZVI, nZVI/GO and J-nZVI/GO

**Figure 1:**
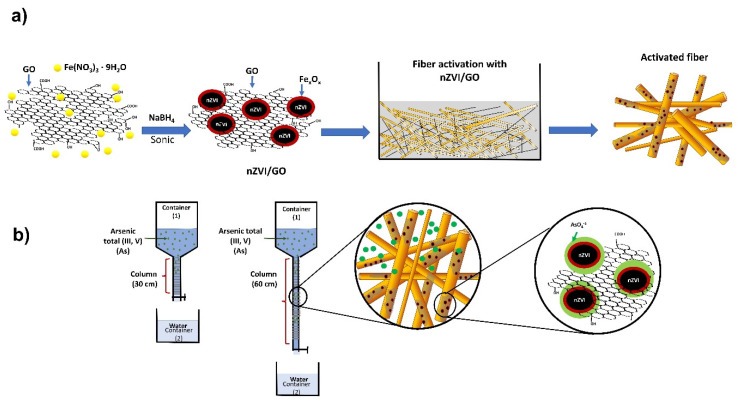
Schematic diagram of (**a**) synthesis of nZVI/GO and activation of jute fibers, (**b**) filtration columns.

The GO was prepared as reported by Rivera et al. [23]. The nanoparticles were prepared by placing 250 mL of Fe(NO_3_)_3_·9H_2_O 0.015 mol/L solution in an ultrasonic bath, bubbling inert gas, for 15 min. Next, 25 mL of NaBH_4_ solution (1.2 mol/L) was added, keeping the bubbling and ultrasonic bath constant. Finally, the nZVI was washed with distilled water three times. The nZVI/GO hybrid was prepared following the same methodology, incorporating GO from the beginning of the preparation (Figure 1a).

### 2.2. Fiber Evaluation: Preparation, nZVI/GO Retention, and Arsenic Adsorption

Fiber preparation: The fiber was washed with soap and distilled water, then dried at room temperature. The dried fiber was immersed in a 0.1 mol/L NaOH solution for 24 h. Afterward, it was washed with distilled water and dried at room temperature.

Retention of nZVI/GO on the fiber: to determine the retention of nZVI/GO on the fiber, 0.5 g of jute fiber was used and impregnated under seven conditions, 0.1, 0.25, 0.5, 1, 2, 3, and 4 mL of nZVI /GO dispersed in water, with a concentration of 14 mg/mL, the final volume is completed to 25 mL with distilled water. It was left to stand for 36 h with shaking. Subsequently, they were rinsed and dried at room temperature. The nZVI/GO retained by the fibers was evaluated as a function of the concentration of the impregnation solution.

Arsenic adsorption: The fibers prepared by the previous step are placed in a 125 mL Erlenmeyer flask with 50 mL of well water at 120 rpm in an orbital shaker for 3 h. Finally, the resultant solution is filtered through 0.2-micron membranes and analyzed by ICP-OES.

### 2.3. Removal Test in a Dynamic Flow

For the removal test (Figure 1b), 150 mL well water was emptied into a container located at the top of the adsorption column (container 1); the filtered liquid was collected in aliquots (container 2) and was analyzed by ICP, the fibers were analyzed by FTIR and SEM.

In the first stage, the filtering time was tested using 1.6 and 4 g of fiber activated with nZVI/GO in a 60 cm long column (J-nZVI/GO-1.6, J-nZVI/GO-4, respectively) and 150 mL of contaminated water.

In the second stage, the amount of fiber was increased to 6 g using the treated fiber in 60 and 30 cm filtration columns (J-60 and J-30) and activated fiber in 60 and 30 cm columns (J-nZVI /GO-60 and J-nZVI/GO-30). The outflow was controlled with a stopcock at the end of the filtration column at 1 mL/min. Additionally, a test was performed using a retention time of 20 min (J-nZVI/GO-60-TR).

In the third stage, the fiber from the previous test was reused after a reactivation treatment. A total of 6 g of this material was tested on each 60 cm filtration column (R-J-nZVI/GO-60) at the same flow rate (1 mL/min). The summary of the test parameters is detailed in Table 1.

## 3. Results and Discussion

### 3.1. Fiber Evaluation: Preparation, nZVI/GO Retention, and Arsenic Adsorption

**Figure 2:**
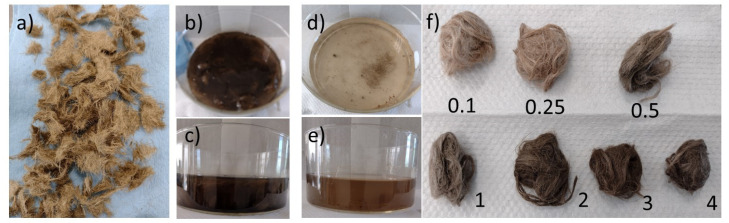
(**a**) Jute fibers washed and treated with NaOH, (**b**,**c**) impregnation of jute with nZVI/GO, (**d**,**e**) residue after impregnation for the 0.25 mL condition, (**f**) aspect of the impregnated fibers.

Figure 2a shows jute fibers washed and treated with NaOH, Figure 2b,c shows the fiber in contact with the solution containing the hybrid nZVI/GO. Figure 2d,e shows the remaining liquid after fiber activation. Although the fiber retains a large part of the hybrid, a significant amount is observed in the residual liquid, this is more evident at high concentrations. Finally, Figure 2f shows the appearance of the fibers as the concentration of the hybrid increases, the hue of the fibers becomes darker because presence of nZVI/GO.

The impregnated material on the fibers will be released by the amount of nZVI/GO in the initial solution (*C*_0_, mg of nZVI/GO) and the residue after activation (*R_e_*, mg of residual nZVI/GO). The result of this difference is expressed by *Q_e_*, which represents the mg of nZVI/GO retained in the fibers, see Equation (1).

(1) $$Q_{e}=C_{0}-R_{e}$$

The retention percentage was determined by following Equation (2).

(2) QeC0×100

**Figure 3:**
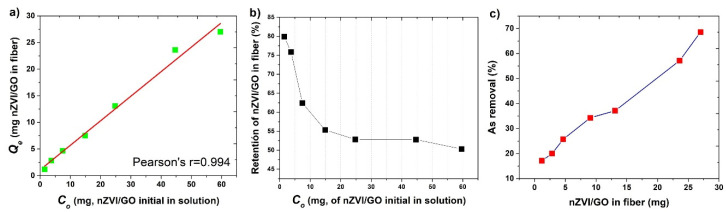
(**a**) Correlation between the initial concentration of nZVI/GO in the solution (*C*_0_) and the amount of material retained on the jute fibers (*Q_e_*). (**b**) the percentage of retained nZVI on the fiber concerning the initial amount added to the activation solution, (**c**) removal percentage of As from the fibers with varying mg of activation.

As shown in Figure 3a, there is a linear correlation between *C*_0_ and *Q_e_* in the range of 1.49 and 59.6 mg, showing that as the initial amount of nZVI/GO in the solution increases, its retention in the fibers also increases. A more evident behavior is observed in the retention percentage (Figure 3b). The retention percentage for amounts as low as 1.49 mg is 79.8% of nZVI/GO in the fiber. For high concentrations such as 14.9 mg, only 50.3% is retained. As *C*_0_ increases, the retained percentage increases in a different proportion. Even when the increase in *C*_0_ reflects an increase in *Q_e_*, the percentage retention does not increase proportionally. Therefore, the percentage retention reaches a saturation limit, which could be taken as the optimum retention point of nZVI/GO on the fiber and would be between 9 and 13 mg of nZVI/GO for every 0.5 g of fiber. Figure 3c shows the As retention percentage on fibers with the nZVI/GO hybrid, which were in contact with 50 mL of the groundwater sample, observing a linear behavior such as that shown when the hybrid is tested alone.

#### 3.1.1. Morphological Analysis

**Figure 4:**
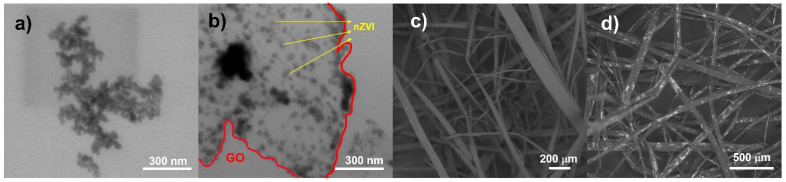
SEM micrographs of (**a**) nZVI nanoparticles by STEM, (**b**) nZVI supported on GO (nZVI/GO), (**c**) natural jute fiber, (**d**) jute fiber activated with nZVI/GO, the white areas indicate the presence of the nZVI/GO hybrid.

Figure 4a shows the individual nZVI nanoparticles with sizes between 17–20 nm. These nanoparticles have a spherical morphology, they form agglomerates due to their high surface energy [22]. In the nZVI/GO, the nanoparticles are observed dispersed and distributed on the GO sheet. The size of the supported nZVI varies from 10 to 20 nm. Few agglomerates can be observed and attributed to a high concentration of metal salt precursors as shown in Figure 4b. Most of the material is homogeneously distributed on the GO surface [24]. Figure 1d and Figure 4c show the morphology of jute fibers, the thickness of the fibers is below 100 microns, presenting a homogeneous surface free of impurities. Figure 4d shows the fibers after activation with the nZVI/GO. A significant difference can be observed in the contrasts of each micrograph before and after activation. These changes are attributed to the change in molecular weight between the natural fiber and that of the particles at the surface of the activated fiber. White or brighter areas on the fiber surface indicate the presence of nZVI/GO distributed on the surface.

**Figure 5:**
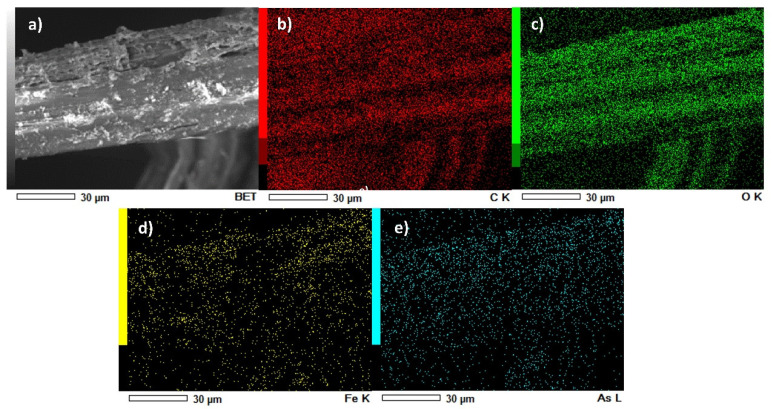
(**a**) Activated jute fiber after the filtration process. (**b**–**e**) Map of element distribution in jute fiber activated by SEM-EDS. Red: carbon, green: oxygen, yellow: iron, and blue: arsenic.

Figure 5a shows an area analyzed by SEM-EDS showing different contrasts between the fiber and the nZVI/GO hybrid deposited on the surface after the filtration process. nZVI/GO appears lighter than the fibers. Figure 5a shows a typical EDS map obtained when the electron beam focused on a nZVI/GO-activated fiber. The colored images show the specific zones with each element present; carbon and oxygen in Figure 5b,c correspond to the amount of GO and cellulose in the fiber structure, which is 60–70% [25]. The iron element is distributed on jute fibers as well as arsenic (Figure 5d,e).

#### 3.1.2. FTIR Spectroscopy

**Figure 6:**
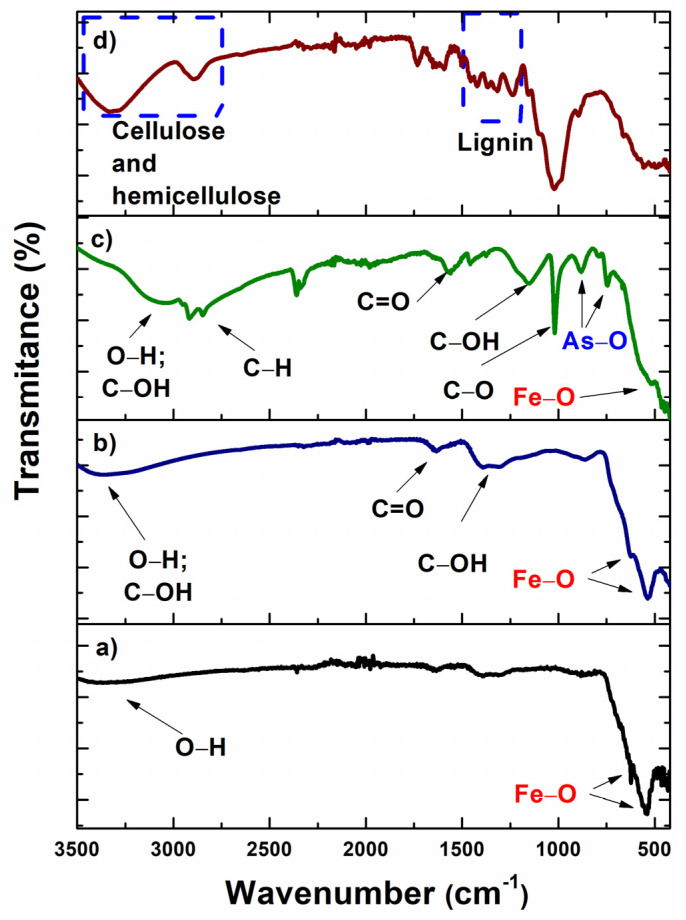
FTIR spectra for (**a**) nZVI, (**b**) nZVI/GO, (**c**) decorated nZVI/GO jute fibers after arsenic ions absorption and (**d**) jute fibers.

The chemical bonds that occur during the J-nZVI/GO hybrid formation, and the adsorption of the total As contaminant, were studied by FTIR spectroscopy in ATR mode (Figure 6). The FTIR spectrum of nZVI nanoparticles (Figure 1a) shows two prominent bands at 542 and 621 cm^−1^ that correspond to a Fe–O stretch inflection [26,27]. A broad, flattened peak at 3450 cm^−1^ denotes the O–H stretching vibration. In addition, a small band at 1625 cm^−1^ is associated with O–H bending [27]. For nZVI/GO (Figure 1b), it is observed that the signal from 3000 to 3500 cm^−1^ increases due to the remaining O–H and C–OH groups of GO. Other characteristic bands of GO are C=C at 1600 cm^−1^, C–OH at 1130 cm^−1^, and 1100 cm^−1^ of the C–O groups. It is observed that the signal corresponding to nZVI is weak at 542 cm^−1^. The As adsorption process by the nZVI/GO hybrid shows the addition of two bands at 740 and 870 cm^−1^, which corresponds to the symmetric and asymmetric stretching of the As–O bond of the adsorbed species [5]. Finally, the spectrum of the J-nZVI/GO as ensemble is presented. The peaks from 3100 to 3500 cm^−1^ correspond to O–H and C–OH stretching vibrations. Peaks 2970 and 2923 cm^−1^ are due to C–H stretching vibrations of cellulose and hemicellulose. In the region from 1530 to 1400 cm^−1^ they are due to lignin components [28,29]. Fiber signals are predominant in this spectrum, it can be seen in Table 2.

### 3.2. Removal Test in a Dynamic Flow: Testing Batch

Removing aqueous contaminants by employing adsorption columns is an attractive technique because it allows continuous and controllable flow operation [30]; however, it represents a more significant challenge for total contaminant removal. The interaction time between the adsorbent material and the arsenic ions is usually lower than in the batch test.

There are a lot of variables in a dynamic flow filtration system, such as length, diameter, column shape, fiber quantity and compaction, flow rate, filtrate volume and time, etc. Due to this, some parameters were fixed: a column diameter of 7 mm, free fall, and controlled flows, a long column of 60 cm to obtain light compaction, and 30 cm for better fiber compaction. This last aspect is essential; the flow is affected depending on the fiber compaction degree. Therefore, the interaction time between the contaminant and the adsorption material can increase or decrease. In batch tests, the adsorption kinetics between nZVI and As require 15 to 20 min of contact for adsorption to be achieved [19,31].

**Figure 7:**
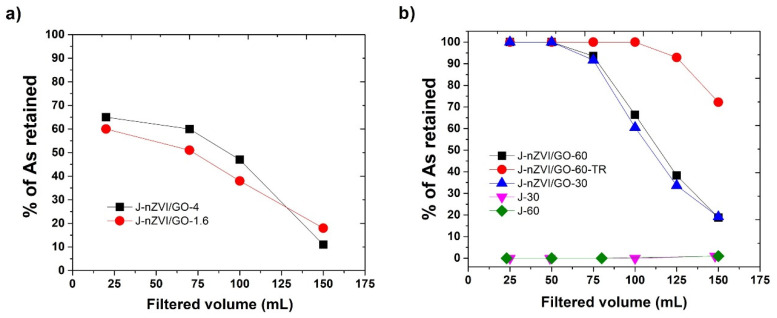
(**a**) Arsenic retention percentage using 1.6 and 4 g of fibers with nZVI/GO, (**b**) arsenic retention percentage in natural fiber and activated fiber in a column with 30 and 60 cm (labels: J-30, J-60, J-nZVI/GO-30, and J-nZVI/GO-60) and a time retention of 20 min (label: J-nZVI/GO-60-TR).

In the first stage, the flow and total removed As were analyzed for the amount of activated fiber within the 60 cm column with a flow in free fall. The J-nZVI/GO-1.6 sample, with 1.6 g of fiber, presented a filtrate flow of 113 mL/min, a fast filtrate rate attributed to the amount of fiber in the column. The J-nZVI/GO-4 sample, with 4 g of fiber, presented a filtration rate of 8 mL/min. This behavior was expected since the filtrate flow depends on the saturation of the column. Both behaviors show that a lower flow allows the As to interact with the nZVI/GO for a longer time, and adsorption of the contaminant on the sample’s surface can occur. At a longer time, the analysis will diffuse through the adsorbate and will interact with all the active groups responsible for adsorption. After 20 mL filtration, sample J-nZVI/GO-4 removed 65% of As, and 60% for the test with the J-nZVI/GO-1.6 sample (Figure 7a). Subsequently, after 70 mL filtration, the removal percentage dropped to 60 and 51% for J-nZVI/GO-4 and J-nZVI/GO-1.6, respectively. Finally, after 150 mL filtration, the removed percentage was 11% for J-nZVI/GO-4 and 18% for J-nZVI/GO-1.6. Due to the fact that total As adsorption depends on the active surface area of the nZVI nanoparticle [32,33], a longer interaction time is required for all the reaction mechanisms between As and nZVI to take place, so the results are consistent with what is reported in the literature for nZVI [19,20].

In the second stage, the amount of fiber was set to 6 g, and two column lengths, 60 and 30 cm, were used to increase compaction (samples were labeled as J-30, J-60, J-nZVI/GO-30, and J-nZVI/GO-60, respectively). A stopcock was used to control flow at the end of the column and maintain a flow of 1 ml/min; this allowed us to take aliquots every 25 mL and also allowed interaction time between As and the fiber with nZVI/GO. Finally, an additional test was conducted where the contaminated water was in contact with the adsorbent system 20 min before filtration (J-nZVI/GO-60-TR).

The results of the second stage are shown in Figure 7b. For the J-30 and J-60 tests, which contain only the treated fiber, there was no arsenic retention beyond 0.5 or 1%. This result shows that contaminant retention, responsible for the removal of the analyte in the filtration system, is completely due to the presence of nZVI/GO. The J-nZVI/GO-60 and J-nZVI/GO-30 tests are similar. The aliquots taken at 25 and 50 mL showed results below the detection limit of ICP-OES equipment (1–5 µg/L). For comparative purposes, it was adjusted to zero, with a total withholding percentage of 100% for these aliquots. For 75 mL of filtered volume, the retention percentage dropped to 93 and 91%, respectively. From 100 mL on, a pronounced drop in retention percentage begins, which is attributed to the saturation and inactivation of the nZVI surface by the presence of As. After 150 mL of filtered solution, the retention percentage was observed at 19 and 18%. Subsequently, tests in columns at 30 and 60 cm were performed with a controlled filtration flow at 1 mL/min. The controlled flow allows the contaminant to remain in contact with the nZVI/GO simultaneously, so a controlled flow could have equivalent results in a column length of 30 or 60 cm with the same fiber content. This effect was observed in the retention percentage of 100% in the aliquots of 25, 50, 75, and 100 mL. In the 125 mL aliquot, the retention percentage decreased to 92%. Finally, after the 150 mL of filtrate, the retention percentage was 72% in the aliquot.

### 3.3. Fiber Reactivation

The fibers used in the previous tests were reactivated using a 2 mol/L NaOH solution for 3 h at 50–60 °C [33,34]. Then, they were impregnated again with the same activation conditions of the fibers. Once dry, we used them to fill the 60 cm column; subsequently, filtration was carried out.

**Figure 8:**
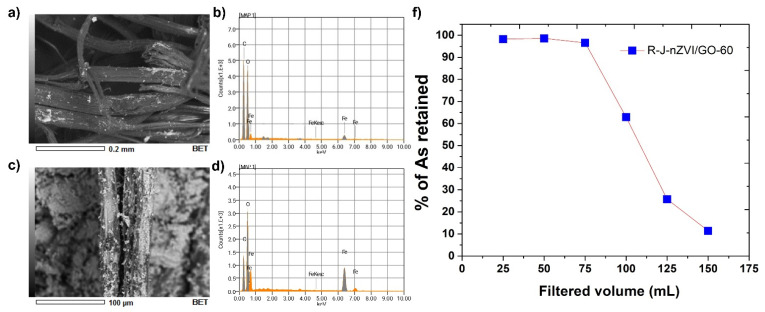
SEM micrographs and EDS elemental analysis, (**a**,**b**) RJ-nZVI/GO-60 test, (**c**,**d**) RJ-nZVI/GO-30, (**f**) retention percentage of the RJ-nZVI/GO-60 test.

SEM micrographs and EDS spectra show the fiber after NaOH treatment (Figure 8a). The points are related to the Fe presence retained by the fiber after treatment. The relative intensity of the existence of Fe is smaller than peak intensity of carbon or oxygen present. The EDS spectra (Figure 8b) show the presence of carbon, oxygen, and iron and the absence of arsenic.

EDS analysis (Figure 8) shows an increase in the Fe signal similar to the carbon intensity. In Figure 8d, we can observe the fibers after reactivation with nZVI/GO. The reactivated fiber was taken to the test bench in a 60 cm column (R-J-nZVI/GO-60) with a filtration flow of 1 mL/min. The R-J-nZVI/GO-60 filtration test is shown in Figure 8f, where the first 25 mL filtration showed 98.2% retention of the total As. For volumes 50 and 75 mL, 98.5 and 96.2% retention were obtained, respectively. Later, a decrease to 25 and 11% retention was observed at 125 and 150 mL of filtrate volume, respectively, presenting an increase in the retention percentage concerning the filtrate volume. Comparing this behavior with the J-nZVI/GO-60 sample under the same test conditions, it is observed that the results are very similar; that is, the fiber used can be reactivated for subsequent filtrations [35].

## 4. Conclusions

The results presented in this work show the synergy between jute fibers and the hybrid nZVI/GO as a system for the adsorption of arsenic present in polluted waters, as well as the importance of a controlled but continuous flow to ensure the maximum adsorption on the surface of the fibers activated with nZVI/GO. The retention percentage of As was 100% before fiber saturation for J-nZVI/GO-60. The fibers used in the first filtration process were reactivated to study their possibility of reuse, demonstrating promising results with a retention percentage above 95%. The studied system promises to be profitable, benefiting non-industrialized sectors, such as communities that do not have the infrastructure to treat contaminated groundwater.

The results presented in this document and future studies will be used to improve the filtration system in preparation for developing an easy-to-use sanitization devise. The reported parameters will also be a base for studying other polluting species in groundwater.

## Figures and Tables

**Table 1:** Test parameters.

Test	Fiber Weight (g)	Flow	Colum Length (cm)
J-nZVI/GO-1.6	1.6	free	60
J-nZVI/GO-4	4	free	60
J-30	6	Controlled	30
J-60	6	Controlled	60
J-nZVI/GO-30	6	Controlled	30
J-nZVI/GO-60	6	Controlled	60
J-nZVI/GO-60-TR	6	controlled + Retention time	60
Reactivated fiber			
RJ-nZVI/GO-60	6	controlled	60

**Table 2:** With FTIR vibrations at different wavelength numbers.

Bond	Vibration	Wavelength Number (cm^−1^)	Ref.
Fe-O	Stretching	540 and 620 1650–1624	[5,24,25,26,27]
O-H	Stretching	3000–3700	
O-H	Bending	1636	
C=C	Stretching	1600–1613	
C-H	Flexion Bending	2867 1352	
CH_2_	Bending	1389	
-COOH	Stretching	1712	
C-O-C	Stretching	1036–1004	
As-O	Symmetrical and antisymmetrical stretching	710	

## Data Availability

Not applicable.
